# Luteolin-7-*O*-Glucoside Present in Lettuce Extracts Inhibits Hepatitis B Surface Antigen Production and Viral Replication by Human Hepatoma Cells *in Vitro*

**DOI:** 10.3389/fmicb.2017.02425

**Published:** 2017-12-06

**Authors:** Xiao-Xian Cui, Xiao Yang, Hui-Jing Wang, Xing-Yu Rong, Sha Jing, You-Hua Xie, Dan-Feng Huang, Chao Zhao

**Affiliations:** ^1^Key Laboratory of Medical Molecular Virology, School of Basic Medical Sciences and National Clinical Research Center for Aging and Medicine, Huashan Hospital, Fudan University, Shanghai, China; ^2^Institutes of Biomedical Sciences, Shanghai Medical College, Fudan University, Shanghai, China; ^3^Key Laboratory of Urban Agriculture (South), Ministry of Agriculture, School of Agriculture and Biology, Shanghai Jiao Tong University, Shanghai, China; ^4^Laboratory of Neuropsychopharmacology, College of Fundamental Medicine, Shanghai University of Medicine and Health Science, Shanghai, China; ^5^Shanghai Key Laboratory of Clinical Geriatric Medicine, Huadong Hospital, Shanghai, China

**Keywords:** hepatitis B virus, hepatitis B surface antigen, luteolin-7-*O*-glucoside, reactive oxygen species, mitochondrial membrane potential

## Abstract

Hepatitis B virus (HBV) infection is endemic in Asia and chronic hepatitis B (CHB) is a major public health issue worldwide. Current treatment strategies for CHB are not satisfactory as they induce a low rate of hepatitis B surface antigen (HBsAg) loss. Extracts were prepared from lettuce hydroponically cultivated in solutions containing glycine or nitrate as nitrogen sources. The lettuce extracts exerted potent anti-HBV effects in HepG2 cell lines *in vitro*, including significant HBsAg inhibition, HBV replication and transcription inhibition, without exerting cytotoxic effects. When used in combination interferon-alpha 2b (IFNα-2b) or lamivudine (3TC), the lettuce extracts synergistically inhibited HBsAg expression and HBV replication. By using differential metabolomics analysis, Luteolin-7-*O*-glucoside was identified and confirmed as a functional component of the lettuce extracts and exhibited similar anti-HBV activity as the lettuce extracts *in vitro*. The inhibition rate on HBsAg was up to 77.4%. Moreover, both the lettuce extracts and luteolin-7-*O*-glucoside functioned as organic antioxidants and, significantly attenuated HBV-induced intracellular reactive oxygen species (ROS) accumulation. Luteolin-7-*O*-glucoside also normalized ROS-induced mitochondrial membrane potential damage, which suggests luteolin-7-*O*-glucoside inhibits HBsAg and HBV replication via a mechanism involving the mitochondria. Our findings suggest luteolin-7-*O*-glucoside may have potential value for clinical application in CHB and may enhance HBsAg and HBV clearance when used as a combination therapy.

## Introduction

Hepatitis B virus (HBV) causes transient and chronic infections of the liver. Although an effective preventive vaccine has been available for more than 30 years, more than 350 million individuals worldwide still have CHB (Curtis et al., 2005). Chronically infected individuals are at high risk for cirrhosis and hepatocellular carcinoma (Buendia, 1992). The HBV vaccine elicits almost no production of protective antibodies in patients with CHB (Trepo et al., 2014). Approved treatments for CHB based on interferons (IFNs) or NAs can effectively decrease HBV viremia. However, neither of these monotherapies nor their combination can effectively reduce serum HBsAg, which is a reliable marker of a favorable long-term prognosis (European Association for the Liver, 2012).

Clinically persistence of HBsAg at least 6 months in host is a sign of chronic infection. HBsAg is required for formation of HBV virions and hepatocyte entry, and may also suppress host immune responses to HBV (Op den Brouw et al., 2009; Patient et al., 2009; Chan et al., 2011). Therefore, elimination of persistence excess antigen production in the serum is thought to break the immunosuppression. HBsAg-positivity can be asymptomatic or lead to progressive active liver disease. In chronic HBV, persistent inflammation results in liver cirrhosis or hepatocellular carcinoma in 25% of patients. (Michel et al., 2015). HBsAg antigenaemia is now considered as a novel target of antiviral therapy. A number of compounds and their derivatives have been identified to function as specific inhibitors of HBsAg secretion (Dougherty et al., 2007; Xu et al., 2014). However, few studies have demonstrated these compounds exert prominent effects, and they normally lead to significant cytotoxicity; Therefore, no specific HBsAg secretion inhibitor is suitable for clinical use. However, animal studies have indicated clevudine reduces HBsAg, partially restores host immune responses and improves the efficacy of therapeutic vaccines (Menne et al., 2002). Therefore, new agents need to be identified.

The leafy vegetable lettuce (*Lactuca sativa* L.) is consumed worldwide and available throughout the entire year. Epidemiological studies have shown lettuce consumption prevents a number of diseases, including cancer and diabetes (Cheng et al., 2014; Lin et al., 2014). Lettuce extracts have been reported to exert anti-oxidative effects. Reactive oxidative species (ROS) are small molecules generated as by-products of normal cellular metabolism of oxygen and also produced in aerobic organisms as a result of enzymatic reactions in various intracellular components, mainly the mitochondria, peroxisomes and ER (Choi and Harhaj, 2014). Significant evidence indicates HBV infection induces a pro-oxidative status. Typical ROS products, several of which are produced during HBV infection, are considered potential carcinogens (Adam-Vizi and Chinopoulos, 2006). In fact, increased level of oxidative stress, sulfhydryl and lipid peroxidation were observed in CHB patients (Duygu et al., 2012). The antioxidant, like NAC were found that they could restrain HBV replication by a mechanism independent of the intracellular level of ROS (Weiss et al., 1996). Recently, Fisicaro et al. (2017) compared the transcriptome profiles of HBV-specific CD8+ T cells from patients with acute and chronic disease with those of HBV-specific CD8+ T cells from patients whose HBV infection spontaneously resolved and influenza-specific CD8+ T cells from healthy participants. Exhausted HBV-specific CD8+ T cells were markedly impaired at multiple levels and showed substantial downregulation of genes involved in various cellular processes that centered on extensive mitochondrial alterations (Fisicaro et al., 2017). A notable improvement of mitochondrial and antiviral CD8+ T cells functions was elicited by mitochondrion-targeted antioxidants, which suggested a central role of ROS in T cell exhaustion in CHB.

Based on this evidence, we hypothesized lettuce extracts, a natural source of organic antioxidants, could decrease HBsAg and inhibit HBV replication by reducing the levels of intracellular excess ROS and altering the intracellular redox state. To test this hypothesis, the effects of the lettuce extracts on HBsAg and HBeAg secretion, HBV replication and transcription were detected in three HBV-expressed HepG2 cell lines. Meanwhile, the intracellular ROS and mitochondrial membrane potential were assessed.

This study demonstrates both lettuce extracts and luteolin-7-*O*-glucoside, which we identified as a functional component of the lettuce extracts, exert potent anti-HBV effects and effectively reduce HBsAg secretion. Moreover, we provide evidence that the components of lettuce could exert a synergistic anti-HBV effect when combined with the existing antiviral drugs IFNα-2b or NAs.

## Materials and Methods

### Lettuce Cultivation

Lettuce (cv. Lollo Rossa) were hydroponically cultivated in a greenhouse at Shanghai Jiao Tong University, China, as described previously (Yang et al., 2017) in nutrient solution (pH 5.8) containing nitrogen fertilizer as 9 mM NaNO_3_ (S11) or 9 mM glycine (S15) for 4 weeks. All leaves were collected at the end of cultivation, flash frozen in liquid N and frozen at -80°C until further analysis.

### Lettuce Metabolites Profiling

Lettuce extracts were prepared as previously described (Yang et al., 2017). Briefly, 0.2 g samples were ground into a powder in liquid N, extracted in 1 mL methanol/water solution (4:1 v/v), sonicated for 30 min at 25°C, incubated at 4°C for 12 h and centrifuged at 12000 *g* for 10 min; 0.5 mL of supernatant was analyzed. Lettuce metabolite profiling was performed using an ultra-performance liquid chromatography-ion mobility spectrometry-quadrupole-time of-flight mass spectrometry (UPLC-IMS-QTOF/MS, Waters Corp., Milford, MA, United States) as described by Yang et al. (2017).

### Cell Culture, Plasmids, Drugs and Reagents

The HBV-producing cell lines HepG2.2.15, HepAD38 and parental cell line HepG2 used for transient transfection were maintained in DMEM supplemented with 10% FBS, 2 mM L-glutamine, 100 IU^.^ml^-1^ penicillin, 100 mg^.^ml^-1^ streptomycin in a humidified atmosphere with 5% CO_2_ at 37°C. DMEM, FBS, L-glutamine, penicillin, and streptomycin were purchased from Gibco Life Technologies (Grand Island, NY, United States).

The p1.3HBV plasmid containing a terminally redundant (1.3-fold length of HBV genome) replication-competent HBV genome was constructed in our lab. Luciferase reporter vectors containing HBV regulatory elements (ENI/Xp, nt1237-1375; ENII/Cp, nt1648–1853; Sp1, nt2224–2784; Sp2, nt2814–3123) were constructed as described previously (Zhang et al., 2011). Recombinant human IFNα-2b was purchased from Sino Biological Inc. (LuDong Area, BDA, Beijing). 3TC purity (>98%) was purchased from Sigma (St. Louis, MO, United States). NAC was purchased from Yeasen Biology Co., Ltd. (Shanghai, China).

### Cell Transfection

HepG2 cells were seeded in 6-well plates at a density of 10^6^ cells per well and transiently transfected with 2 μg of plasmid DNA using Fugene HD (Promega, Madison, WI, United States) following the manufacturer’s instructions.

### Treatment with Lettuce Extracts and Other Antiviral Agents

Prior to the *in vitro* assays, 0.5 mL of lettuce extracts was vacuum freeze-dried at 25°C and resuspended in 200 μL water. HepG2.2.15 cells were seeded in 6-well plates at a density of 10^6^ cells per well, cultured for 24 h, then the lettuce extracts, 3TC and/or IFNα-2b were added at the concentrations indicated in the figures. The media containing drugs were changed every 2 days. Cells were harvested at day 5 after plating. For p1.3HBV-transiently transfected HepG2 cells, 10^6^ cells were seeded per well, treated and harvested after 96 h treatment.

### CCK-8 Assay

The viability of three cell lines we used were determined using the CCK8 assay (Dojindo, Kumamoto, Japan). Cells were seeded at a density of 5000 cells per well in 96-well plates, incubated for 24 h, then treated with different concentrations of lettuce extracts for 2 days. The control wells contained an equivalent amount of medium. All treatment groups had five replicates. CCK8 regent was added to each well, incubated for 1 h, and absorbance values were determined using a microplate reader (Bio-Rad, Hercules, CA, United States) at 450 nm.

### Assessment of HBsAg, HBeAg, and HBV DNA

The levels of HBsAg and HBeAg in the culture supernatants were assessed in a semi-quantitative manner using ELISA kits (Kehua, Shanghai, China) against a standard curve. Absorbance was measured at 450 and 630 nm using a microplate reader (Bio-Rad). Initially, the standard curves were validated against commercial quantitative assays (Adicon, Shanghai, China) performed in parallel (**Supplementary Figure S1G**).

Intracellular HBV DNA was measured in a semi-quantitative manner by Q-PCR on an ABI 7500 Real-Time PCR System (Applied Biosystems, Foster City, CA, United States) using SYBR Green QPCR Master Mix (Promega, Madison, WI, United States) according to the manufacturer’s instructions. The DNA was quantified using specific primers: HBV-Core-F (5′-CCTTCTTACTCTACCGTTCC-3′), HBV-Core-R (5′-GACCAATTTATGCCTACAGCC-3′). All assays were performed two times independently, each in triplicate repetition when tested.

### Southern Blot Detection of HBV Replication Intermediates

Southern blot hybridization was used for the detection of intracellular HBV RI and performed as described previously (Bai et al., 2016). The assay was carried out two times independently.

### RNA Isolation and Reverse Transcript Q-PCR Analysis

Total RNA was isolated from cells using TRIzol reagent (Invitrogen, Carlsbad, CA, United States) according to the manufacturer’s protocol, and the RNA was used for cDNA synthesis from 40 ng total RNA with PrimeScript RT kit (Takara, Shiga, Japan). Primers HBV2270F (5′-GAGTGTGGATTCGCACTCC-3′) and HBV2392R (5′-GAGGCGAGGGAGTTCTTCT-3′) were used for HBV 3.5 kb transcripts (123 bp fragment); HBV1805F (5′-TCACCAGCACCATGCAAC-3′) and HBV1896R (5′-AAGCCACCCAAGGCACAG-3′) were for total HBV-specific transcripts (92 bp fragment). Real-time PCR was conducted by denaturation at 95°C for 30 s, followed by 40 cycles of 95°C denaturation for 3 s, and 60°C annealing/elongation for 30 s using SYBR QPCR Master Mix (Promega, Madison, WI, United States) on an ABI 7500 Real-Time PCR System. The assays were carried out two times independently.

### Northern Blot of HBV RNA Transcripts

Northern blot hybridization was used for the detection of intracellular HBV RNA transcripts as described previously (Lin et al., 2017). The assay was carried out two times independently.

### ROS Assays

The intracellular superoxide anion levels were measured using DCFH-DA (Yeasen Biology Co., Ltd., Shanghai, China) by Fluorescence Activated Cell Sorting (FACS). Mitochondrial superoxide production in the live cells was detected using the MitoSOX Red assay (Life Technologies, Grand Island, NY, United States) on a FACScan flow cytometer (BD Biosciences, Franklin Lakes, NJ, United States). The experiments were repeated three times.

### Measurement of Mitochondrial Membrane Potential by Laser Scanning Confocal Microscopy Using Mitored

Cells cultured at low density on fibronectin-coated 35 mm glass-bottomed dishes were incubated for 20 min at 37°C with MitoRed probe (Dojindo) to assess mitochondrial membrane potential and DAPI to locate nuclei. Stained cells were washed with PBS and examined using a Laser Scanning Confocal microscope (Leica, Wetzlar, Germany). The experiments were repeated two times.

### Measurement of Mitochondrial Membrane Potential by FACS Using JC-1

At 2 h after treatment, the cell culture medium was removed, cells were digested with trypsin and single cell suspensions were prepared and incubated with JC-1 dye (Yeasen Biology Co., Ltd., Shanghai, China) for 20 min at 37°C in the dark. Cells were washed with PBS and immediately analyzed using a FACScan flow cytometer. A total of 10,000 cells were examined; green fluorescence was monitored using a 529 nm filter and orange fluorescence using a 590 nm filter. The experiments were repeated three times.

## Results

### Lettuce Extracts Suppress HBV Antigen Secretion in HBV-Expression Cells

**FIGURE 1:**
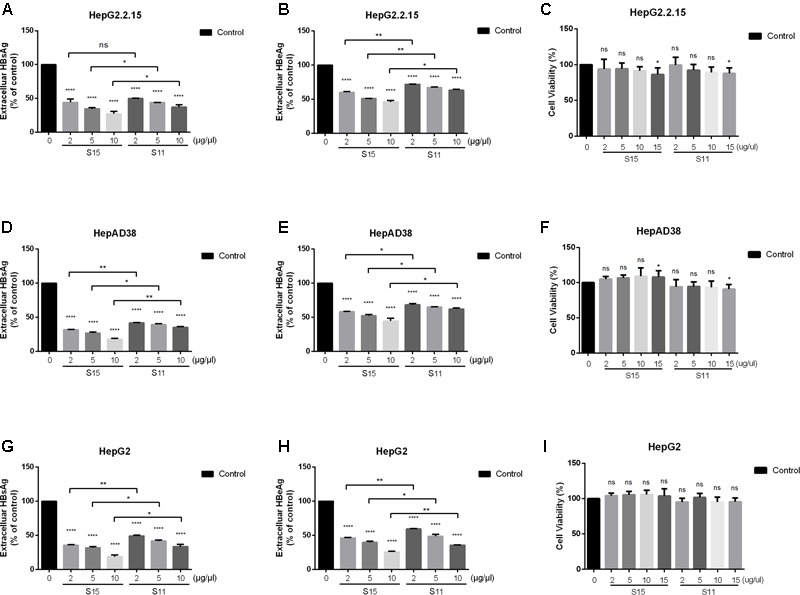
Inhibitory effect of lettuce extracts on HBsAg and HBeAg expression in HepG2.2.15 cells, HpeAD38 cells and HepG2 cells transiently transfected with a replication-competent HBV clone. Cells were treated with two groups of lettuce extracts hydroponically cultured with different nitrogen source (S15 treated with 9 mM glycine and S11 with the same concentration of nitrate) at the indicated concentrations for 2 days. Dose-dependent suppression of HBV antigen expression by lettuce extract in different HBV cell lines **(A,B,D,E)** and HepG2 cells transiently transfected with HBV **(G,H)**. HBsAg and HBeAg were quantified using an electrochemical illuminescent immunoassay. The CCK8 assay was used to determine the cytotoxic effect of lettuce extracts in different cell lines **(C,F,I)**. All values are expressed as percentages relative to untreated control (Control). Statistical significance was calculated using student’s *t*-test. Ns, not significant, *^∗^P* < 0.05, ^∗∗^*P* < 0.005, ^∗∗∗^*P* < 0.0005, ^∗∗∗∗^*P* < 0.0001.

The effects of the two lettuce extracts cultivated by 9 mM NaNO3 (S11) and 9 mM glycine (S15) on expression of viral antigens were investigated by measuring the rate of HBsAg and HBeAg secretion by HBV-expressing cells. Both lettuce extracts significantly suppressed HBsAg and HBeAg secretion compared with vehicle control cells (**Figures 1A,B,D,E,G,H**). The maximum rate of HBsAg secretion inhibition was 82.5% in HepAD38 cells.

To exclude the possibility that the reduced antigen secretion in lettuce extract-treated cells was due to a cytotoxic effect, cell viability was quantified using the CCK8 assay. No cytotoxic effects were observed in all the three cell lines until the concentration was up to 15 μg/μl, so we choose 10 μg/μl as the maximum concentration in our further exploration (**Figures 1C,F,I**).

### Lettuce Extracts Suppress HBV Replication and Transcription

**FIGURE 2:**
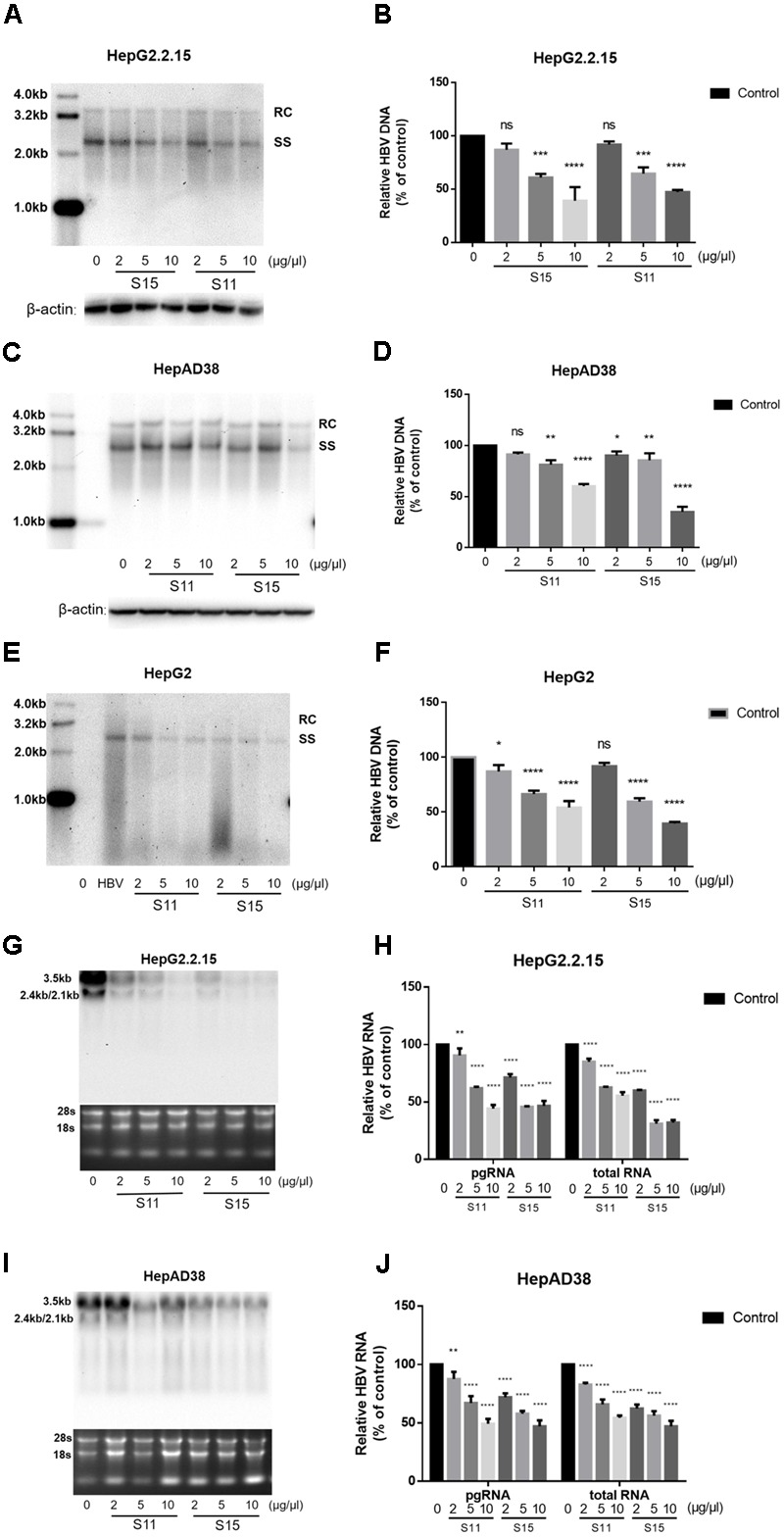
Lettuce extracts S11, S15 suppresses HBV replication in a dose-dependent manner in HepG2.2.15 cells, HpeAD38 cells and HepG2 cells transiently transfected with HBV. HBV replication intermediates were extracted and detected by southern blot hybridization **(A,C,E)** and quantified using fluorescence quantitative PCR **(B,D,F)**. The positions of relaxed circular (RC) and single-stranded (SS) DNA forms are indicated. Viral RNAs were examined by Northern blot **(G,I)** and quantified using fluorescence quantitative PCR **(H,J)**. All values are expressed as percentages relative to untreated control. RC, relaxed circular DNA; SS, single-stranded DNA. β-actin served as the DNA loading control and 18S/28S RNAs served as the RNA loading control. Statistical significance was calculated using student’s *t*-test. Ns, not significant, ^∗^*P* < 0.05, ^∗∗^*P* < 0.005, ^∗∗∗^*P* < 0.0005, ^∗∗∗∗^*P* < 0.0001.

Subsequently, the effects of the lettuce extracts on HBV replication and transcription were determined. As shown in **Figures 2A–D,G–J**, treatment with the lettuce extracts significantly decreased intracellular HBV DNA and RNA levels in HepG2.2.15 and HepAD38 cells. The maximum inhibition rate was 35% for HBV DNA and 43.9% for viral RNA. Similar results were observed in HepG2 transiently transfected with HBV (**Figures 2E,F**).

**FIGURE 3:**
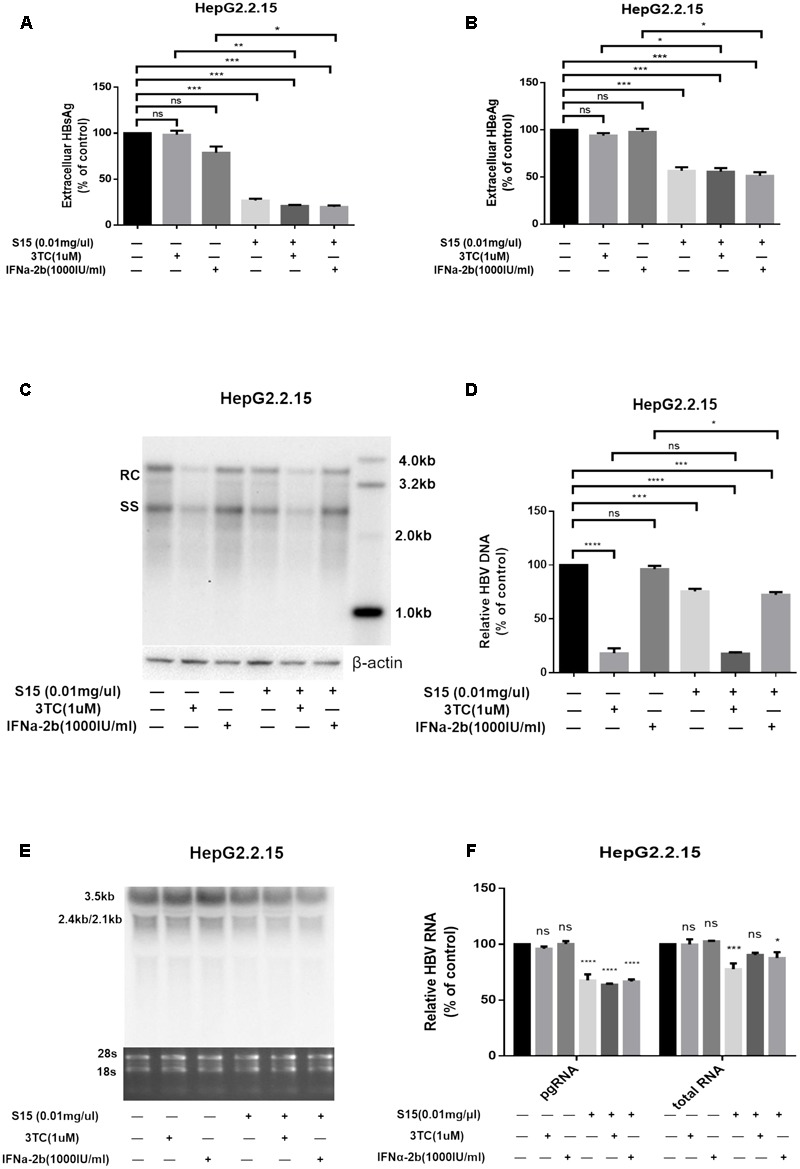
Lettuce extracts S15 suppresses HBV viral antigen secretion **(A,B)**, replication **(C,D)** and transcription **(E,F)** in the absence or presence 3TC and IFNa-2b in HepG2.2.15 cells. HBsAg and HBeAg levels in culture supernatants were measured using an electrochemical illuminescent immunoassay **(A,B)**. HBV replication intermediates were extracted and detected by Southern blot hybridization **(C)** and quantified using fluorescence quantitative PCR **(D)**. Viral RNAs were examined by Northern blot **(E)** and quantified using fluorescence quantitative PCR **(F)**. All values are expressed as percentages relative to untreated control. RC, relaxed circular DNA; SS, single-stranded DNA. β-actin served as the DNA loading control and 18S/28S RNAs served as the RNA loading control. Statistical significance was calculated using student’s *t*-test. Ns, not significant, ^∗^*P* < 0.05, ^∗∗^*P* < 0.005, ^∗∗∗^*P* < 0.0005, ^∗∗∗∗^*P* < 0.0001.

Next, we tested the combined effects of the lettuce extracts and 3TC or IFNα-2b. HepG2.2.15 cells were reported to be insensitive to the treatment with interferon α-2b, IFNα-2b measurably inhibited HBV replication in the HepG2.2.15 cells only at high concentrations (normal IC_50_ about 3000–4000 IU/ml) (Sun and Nassal, 2006), in which it showed apparent toxic effect. The concentration of IFNα-2b we used in **Figure 3** was 1000 IU/ml to keep the cell status. Combination of the lettuce extracts with 3TC and/or IFN led to more significant reductions in HBV antigen expression and replication in HepG2.2.15 compared to cells treated with 3TC or IFNα-2b alone (**Figures 3A–F**).

The higher concentration (4000 IU/ml) of IFNa-2b in HepG2.2.15 cell line was used, and the result was shown in the **Supplementary Figure S2**. At the same time, we supplemented the same assay in HepAD38 cell line, and the results were added into **Supplementary Figure S3**.

### Luteolin-7-*O*-Glucoside Is a Functional Component of Lettuce Extracts

**FIGURE 4:**
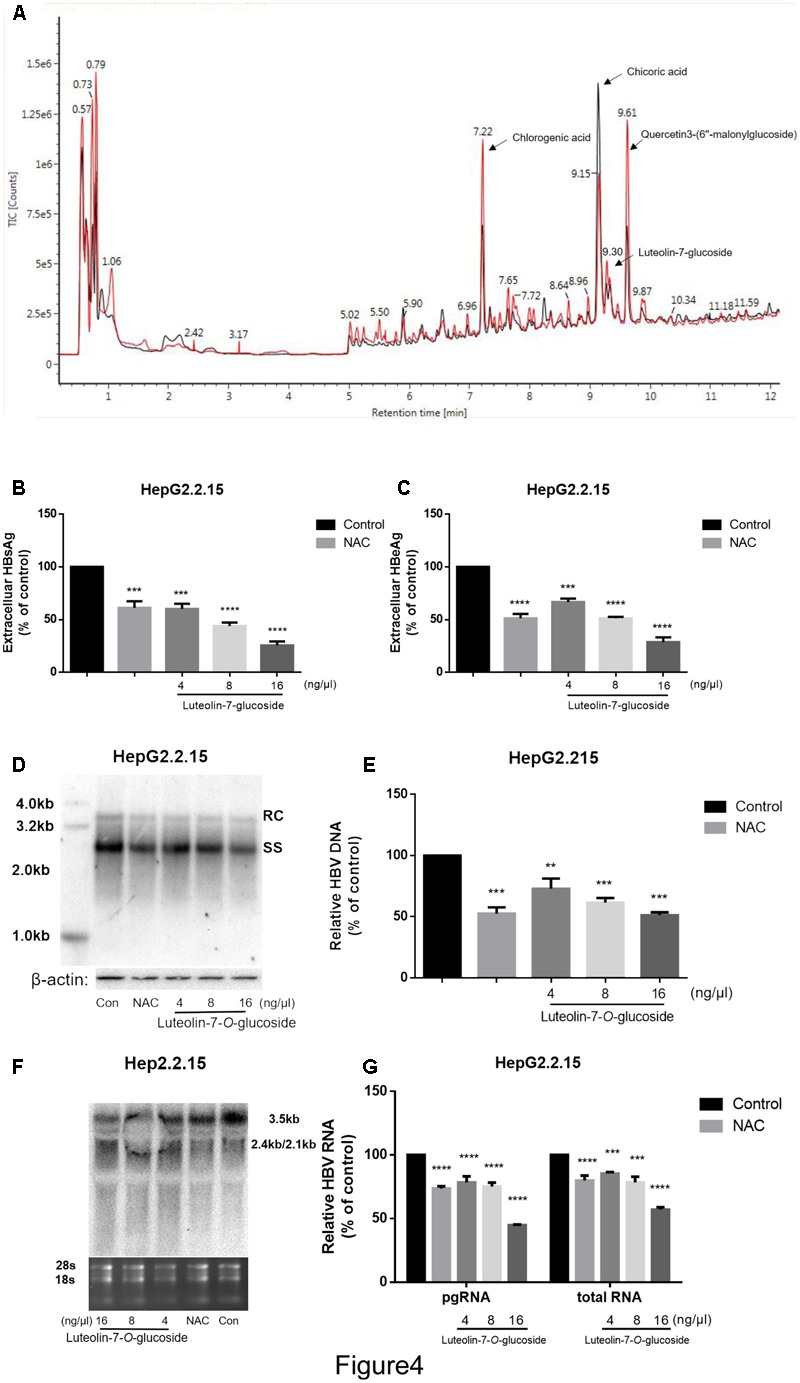
The identification of the main effective components of lettuce extracts and confirmation of its inhibitory effects on HBV. The components of lettuce extracts were determined by UPLC-IMS-QTOF/MS and analyzed by differential metabolomics approach. The red line was different with black line (except for the start because of chromatographic co-effluents) **(A)**. Based on online databases, literature and standards, and the collision cross section values, chlorogenic acid, chicoric acid, luteolin-7-*O*-glucoside and quercetin 3-(6″-malonylglucoside) were identified. The luteolin-7-*O*-glucoside suppresses HBV viral antigen in a dose-dependent manner in HepG2.2.15 cell line and 10 mM NAC was used as a positive control **(B,C)**. HBsAg and HBeAg levels in culture supernatants were measured using an electrochemical illuminescent immunoassay. HBV replication intermediates were extracted and detected by Southern blot hybridization **(D)** or quantified using fluorescence quantitative PCR **(E)**. Viral RNAs were examined by Northern blot **(F)** or quantified using fluorescence quantitative PCR **(G)**. All values are expressed as percentages relative to untreated control. RC, relaxed circular DNA; SS, single-stranded DNA. β-actin served as the DNA loading control and 18S/28S RNAs served as the RNA loading control. Statistical significance was calculated using student’s *t*-test. Ns, not significant, ^∗^*P* < 0.05, ^∗∗^*P* < 0.005, ^∗∗∗^*P* < 0.0005, ^∗∗∗∗^*P* < 0.0001.

The lettuce extracts were assessed by UPLC/IMS/QTOF-MS to investigate their functional composition. As shown in **Figure 4A**, the lettuce extract obtained from the plants cultivated in glycine (red line) was different to the lettuce extract obtained from the plants cultivated in nitrate (black line) at the peak retention times of 7.22, 9.30, 9.15, and 9.61 min. These four major components were identified as chlorogenic acid, luteolin-7-*O*-glucoside, chicoric acid and quercetin-3-(6″-malonyl-glucoside), respectively (*P* < 0.01, *t*-test; fold change between groups >2).

Next, we treated HepG2.2.15 cells with different concentrations of luteolin-7-*O*-glucoside, chlorogenic acid, chicoric acid or quercetin-3-(6″-malonylglucoside) standards to assess their anti-HBV effects. As shown in **Supplementary Figures S1C–E**, quercetin-3-(6″-malonylglucoside), chicoric acid and chlorogenic acid exerted cytotoxic effects, with maximum inhibitory concentrations of 0.016 μg^.^μL^-1^, but not in luteolin-7-*O*-glucoside (**Supplementary Figures 1A,B**). Moreover, the CCK8 assay provided consistent results and large numbers of vacuoles were observed in cells treated with chlorogenic acid, chicoric acid and quercetin-3-(6″-malonylglucoside) (**Supplementary Figure S1F**). However, no significant inhibition of cell viability or proliferation were observed in cells treated with luteolin-7-*O*-glucoside at an equivalent concentration that induced the same inhibitory effects as the lettuce extracts, which indicates luteolin-7-*O*-glucoside plays an important role in the non-cytotoxic HBV-suppressive effect of lettuce extracts.

Additionally, the levels of HBsAg and HBeAg in the media were determined after treatment with different concentrations of luteolin-7-*O*-glucoside. And 10 mM NAC was used as a positive control according to the previous study (Weiss et al., 1996). ELISA indicated that luteolin-7-*O*-glucoside efficiently reduced the levels of both HBsAg and HBeAg in the media in a dose-dependent manner (**Figures 4B,C**). The maximum inhibition rate of HBsAg secretion was 75%. Southern blot and northern blot revealed that luteolin-7-*O*-glucoside inhibited the replication and transcription of HBV in a dose-dependent manner (**Figures 4D,F**); quantitative real-time PCR analyses confirmed these results (**Figures 4E,G**). The maximum inhibition rates were 51 and 44.7% for HBV DNA and viral RNA, respectively.

### Luteolin-7-*O*-Glucoside from Lettuce Extracts May Inhibit HBV by Altering Intracellular Redox State and Alleviating Mitochondrial Dysfunction

**FIGURE 5:**
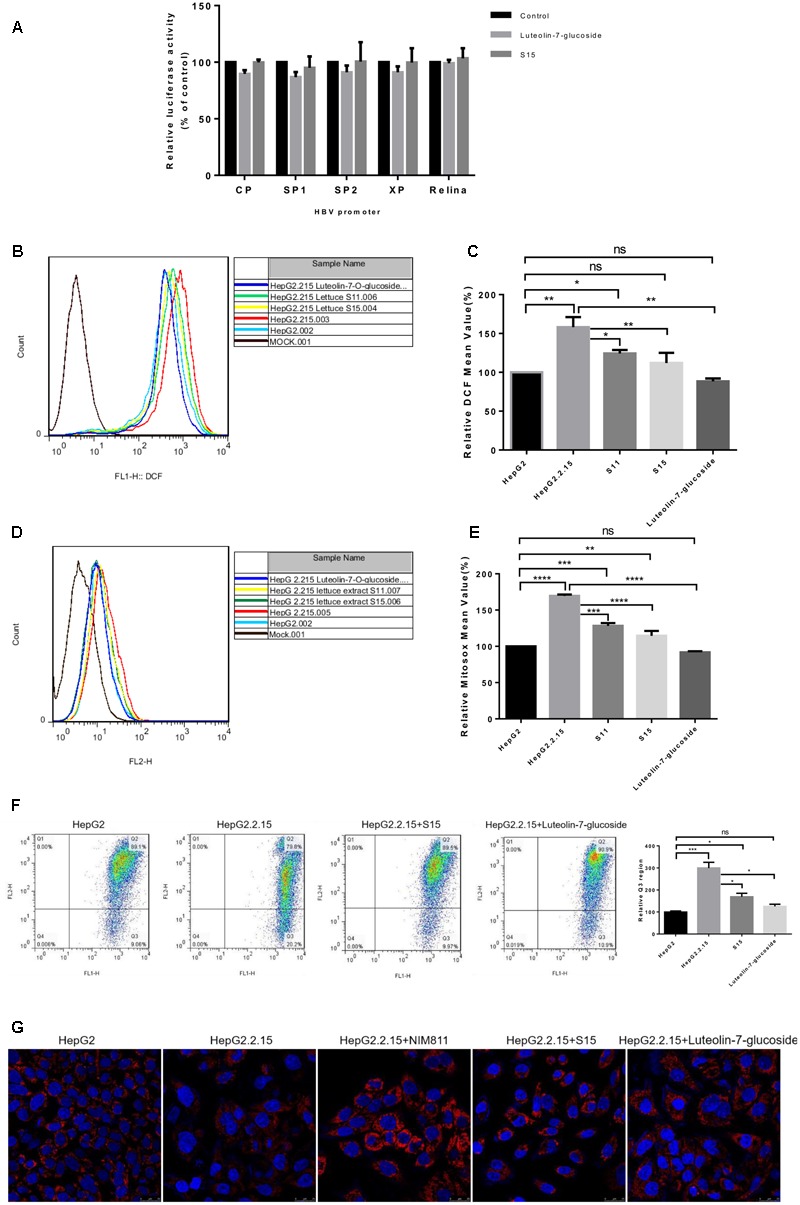
Reactive oxygen species and mitochondrial membrane potential measurement after treatment of HepG2 2.2.15 cells with lettuce extracts and luteolin-7-*O*-glucoside to explore the mechanism of anti-HBV. The lettuce extracts and luteolin-7-*O*-glucoside have no effect on the activities of HBV promoters in HepG2 cells **(A)** and influence intracellular redox **(B,C)** status and mitochondrial membrane potential **(D,E)**. HepG2 cells were transient transfected with the luciferase reporter plasmids with HBV promoters Sp1, Sp2, ENII/Cp, and ENI/Xp. A control of Relina (Promega, Madison, WI, United States) was used to exclude non-specific toxic effects of the treatment. Luciferase activities were determined using the luciferase assay. HBV-producing cell line HepG2.2.15 and HepG2 cell line were stained with DCFH **(B,C)** and MitoSOX Red **(D,E)** probes and ROS levels were determined by FACS. Besides, the mitochondrial membrane potential was quantified by JC-1 dye using FACS **(F)**. Means and SDs of data from three independent experiments are plotted. Detection of mitochondrial membrane potential of HepG2.2.15 using MitoRed dye **(G)** by confocal microscopy. The fluorescence intensity of HepG2.2.15 became stronger after treatment with lettuce extract S15 and luteolin-7-*O*-glucoside. NIM811 could be the positive control of stabilize the mitochondrial membrane potential. All values are expressed as percentages relative to untreated control. Statistical significance was calculated using student’s *t*-test. Ns, not significant, ^∗^*P* < 0.05, ^∗∗^*P* < 0.005, ^∗∗∗^*P* < 0.0005, ^∗∗∗∗^*P* < 0.0001.

To clarify the mechanisms responsible for the anti-HBV effects of lettuce extracts, the transcriptional activities of four HBV promoters in HepG2 cells were examined using a dual luciferase reporter assay. The relative activities of all four HBV promoters (normalized to the control reporter) were almost unchanged after treatment with extracts (**Figure 5A**). This result suggests that ability of the lettuce extracts to reduce viral antigen production, replication and transcription were not strongly associated with down-regulation of HBV promoter activity.

Previous studies indicated HBV replication can be influenced by the cellular redox state and induced by ROS production. As luteolin-7-*O*-glucoside acts as a reducing agent, we assessed intracellular redox state using the DCFH and MitoSOX assays. Intracellular and mitochondrial ROS levels were higher in HepG2.2.15 cells than HepG2 cells and the lettuce extracts and luteolin-7-*O*-glucoside decreased ROS levels in HepG2.2.15 cells (**Figures 5B,D**). The experiments were repeated three times and the **Figures 5C,E** showed the specific statistical results.

High levels of ROS can induce damaging effects, including changes to mitochondrial membrane potential, which plays a central role in oxidative processes. JC-1 (5,5′,6,6′-tetrachloro-1,1′,3,3′-tetraethylbenzimidazolylcarbocyanine iodide) can be used as an indicator of mitochondrial potential in normal cells with high electrical membrane potential (Δψm), JC-1 forms red fluorescent J-aggregates (Q2 region), whereas the sensor is detected as green fluorescent monomers (Q3 region) in cells with depolarized or damaged mitochondria. The Q3/Q2 ratio can be used to measure changes in mitochondrial membrane potential. As shown in **Figure 5F**, the Q3/Q2 ratio was higher in HepG2.2.15 cells than HepG2 cells, and the lettuce extracts and luteolin-7-*O*-glucoside decreased the Q3/Q2 ratio in HepG2.2.15 cells.

Moreover, mitochondrial membrane potential was assessed using MitoRed (9-[2-(4′-methylcoumarin-7′-oxycarbonyl) phenyl]-3,6-bis(diethylamino) xanthylium chloride) staining (**Figure 5G**). The interaction of MitoRed with mitochondria is dependent on mitochondrial membrane potential; MitoRed concentrates in normal mitochondria its fluorescence intensity reflects the change in membrane potential. The MitoRed assays showed that the lettuce extracts and luteolin-7-*O*-glucoside protected mitochondrial membrane potential in cells.

Overall, these findings indicate lettuce extracts, especially the functional component luteolin-7-*O*-glucoside, may reduce HBV-induced mitochondrial ROS production, prevent sustained activation of related signaling pathways and ultimately contribute to suppression of HBV antigens expression and viral replication.

## Discussion

The cure of CHB remains the most challenging issue in hepatitis B infection. HBsAg is potentially relevant to clinical cure of CHB, as clearance of HBsAg may be the important way to break immune tolerance. Reestablish of the immune system is critical for HBV cure. At present, treatment of chronic HBV infection includes antiviral drugs such as NAs and immunotherapies such as IFN. However, while these strategies have acceptable efficacy to reduce HBV viremia, these regimens only lead to HBsAg loss in a minority of HBeAg-positive patients after 12 months’ therapy (7% for IFN; 3% for NAs) (European Association for the Liver, 2012), which is not satisfactory. Moreover, drug resistance and side effects are also obstacles to the treatment of CHB. Here, we demonstrate that a component of lettuce extracts, luteolin-7-*O*-glucoside, may represent a promising therapy for CHB. Both the lettuce extracts and luteolin-7-*O*-glucoside showed antiviral activities, especially pronounced inhibition on HBsAg.

Three cell lines were used to assess the anti-HBV activity of the lettuce extracts *in vitro*: HepG2.2.15 [stably transfected with four 5′–3′ tandem copies of the HBV genome positioned such that two dimers of the genomic DNA are 3′–3′ with respect to one another (Sells et al., 1987)], HepAD38 [stably transfected with a single copy of the cDNA of HBV pgRNA under the control of the tetracycline-responsive cytomegalovirus immediate-early (CMV-IE) promoter, which can produce higher levels of HBV DNA (Ladner et al., 1997)] and HepG2 cells transiently transfected with p1.3HBV. The lettuce extracts efficiently inhibited HBsAg; This effect is very relevant to clinical treatment of CHB, as clearance of HBsAg may be required to break immune tolerance. The lettuce extracts also exerted other antiviral activities, including inhibition of HBeAg, as well as HBV replication and transcription, which merit further exploration.

Whether the combination of IFN with NA improves the rate of off-treatment response and HBsAg seroconversion and shortens treatment duration remains a major clinical question in antiviral therapy for CHB. Despite the fact combining PegIFNα-2b with 3TC or LdT results in a higher on-treatment virological response, this combination does not result in a higher rate of sustained off-treatment virological or serological response (Marcellin et al., 2004; Janssen et al., 2005; Lau et al., 2005). In this study, we demonstrated the lettuce extracts exerted synergistic antiviral effects *in vitro* when combined with 3TC or IFNα-2b. Notably, the lettuce extracts inhibited HBsAg secretion by up to 77 and 81% when combined with 3TC or IFNα-2b, respectively. This striking effect indicates the functional components of lettuce extracts may represent an attractive prospect for the treatment of CHB.

Moreover, HepG2.2.15 cells were insensitive to the treatment with IFNα-2b but the lettuce extract may enhance the sensitivity of cells to IFNα-2b in our system. In addition, such combination approaches may reduce the dosage of drug required, and also reduce drug resistance to NAs and the side-effects of interferons. These effects need to be further investigated.

To identify the functional antiviral components in lettuce extracts, gas chromatography-mass spectrometry (data not shown) and UPLC-IMS-QTOF/MS were used to compare the metabolites in the lettuce extracts obtained from plants hydroponically cultivated in different fertilizers. A number of differences were observed, including amino acids, flavonoids and phenolic acids. Amino acids exerted no significant inhibitory effect on HBV (data not shown). Flavonoids and phenolic acids have been demonstrated to possess anti-viral and anti-inflammatory activities. Therefore, the antiviral effects of the most significantly altered metabolites, including luteolin-7-*O*-glucoside and quercetin-3-(6″-malonylglucoside), chorogenic acid and chicoric acid in the two lettuce extracts were investigated. Phenolic acids (including chlorogenic acid and chicoric acid) and quercetin-3-(6″-malonylglucoside) exerted cytotoxic effects and induced vacuolar changes at high concentrations in cells (**Supplementary Figures S1C–E**). However, these compounds did exert some anti-HBV activity at lower concentrations (data not shown). In contrast, the concentrations of luteolin-7-*O*-glucoside (presented the equivalent antiviral effect as in the lettuce extracts) showed no cytotoxic effect, and significantly inhibited HBV replication and transcription, indicating luteolin-7-*O*-glucoside plays a significant role in the anti-HBV activity of the lettuce extracts. However, we cannot exclude the possibility that the anti-HBV effect of the lettuce extracts may be the result of synergistic interactions between different functional components. This need to be clarified further.

Mechanistically, the HBV promoter assay suggested that the repressive effects of the lettuce extracts and luteolin-7-*O*-glucoside on HBsAg and HBeAg production and viral replication were not mediated via downregulation of HBV promoter activity. Therefore, we assessed whether lettuce extracts and luteolin-7-*O*-glucoside exert anti-HBV effects via another mechanism.

Herbal medicines such as glycyrrhizin have been used for a long time to treat liver disease; these compounds possess hepatocyte protective effects and antiviral activity. However, standardized manufacturing processes and detailed studies are required to confirm the efficacy and mechanism of action of these compounds. Numerous studies have reported several plant extracts possess anti-HBV activity (Xu et al., 2008; Kim et al., 2009; Rechtman et al., 2010; Pei et al., 2011; Huang et al., 2014; Zhang et al., 2016) as well as anti-inflammatory and antioxidant properties. For instance, Pei et al. (2011) reported Pu-erh tea extracts and its components function as reducing substances and reduce the intracellular ROS level induced by HBV integration *in vitro*. An isolation from *Artemisia morrisonensis* specifically reduced ER stress-related GRP78 RNA/protein levels (Huang et al., 2014). Similarly, by comparing the levels of ROS in HepG2 and HepG2.2.15 cells, we found the lettuce extracts and luteolin-7-*O*-glucoside functioned as reducing substances to ameliorate HBV-induced oxidative stress. Moreover, both the lettuce extracts and luteolin-7-*O*-glucoside attenuated HBV-induced mitochondrial dysfunction.

Mitochondria, the powerhouses of the cell that produce up to 90% of ATP, are the major source of intracellular ROS, play a fundamental role in cell fate and function as control point during viral invasion. Unchwaniwala et al. (2016) found HBV polymerase could localize to the mitochondria, and proposed HBV polymerase may alter one or more host mitochondrial functions to gain a replicative advantage and persist in chronically infected individuals. Multiple studies have reported HBV associates with the outer mitochondrial membrane through hepatitis B virus X protein (HBX) and modulates mitochondrial calcium uptake, which in turn regulates cytosolic calcium levels and signaling to drive HBV replication (Bouchard et al., 2001; Clippinger and Bouchard, 2008; Lucifora et al., 2011). CsA and its derivatives NIM811 inhibit HBV replication and HBsAg production *in vitro* by interfering with the mitochondria to block cytosolic calcium signaling (Waldmeier et al., 2002; Xia et al., 2005; Phillips et al., 2015). We found the lettuce extracts and luteolin-7-*O*-glucoside suppressed mitochondrial calcium uptake (data not shown), which may help to regulate cytosolic calcium signaling and inhibit HBV replication. Based on the literature and the data in this study, we hypothesize the antiviral activity of the lettuce extracts and luteolin-7-*O*-glucoside are mainly mediated via mitochondrial alterations. We plan to further explore the interaction between cytosolic calcium signaling and luteolin-7-*O*-glucoside in future studies.

## Conclusion

The luteolin-7-*O*-glucoside present in lettuce extracts exerts a potent anti-HBV effect *in vitro* that may be clinically relevant. Moreover, luteolin-7-*O*-glucoside may work synergistically with current antiviral drugs to improve the treatment of CHB.

## Author Contributions

X-XC, XY, and H-JW performed the experiments, analyzed the data, and drafted the manuscript. X-YR and SJ helped with the figures and samples. Y-HX provided some useful suggestions for the study and manuscript. CZ and D-FH designed and supervised the work, analyzed the data and contributed to writing the manuscript.

## Conflict of Interest Statement

The authors declare that the research was conducted in the absence of any commercial or financial relationships that could be construed as a potential conflict of interest.

## Supplementary Material

FIGURE S1 Cytotoxicity of lettuce extracts and differential metabolites in HepG2.2.15 cells. **(A)** Represented the morphology of normal cells and **(B)** represented the morphology of the cells that treated with luteolin-7-*O*-glucoside. A large number of vacuoles were observed in cells treated with quercetin-3-(6″-malonylglucoside) **(C)**, chicoric acid **(D)** and chlorogenic acid **(E)** at the concentrations. The CCK8 assay provided consistent results as the morphological analysis **(F)**. Semi-quantitative analysis of HBsAg and validation of the HBsAg ELISA standard curve using a commercial quantitative assays **(G)**. Statistical significance was calculated using student’s *t*-test. Ns, not significant, ^∗^*P* < 0.05, ^∗∗^*P* < 0.005, ^∗∗∗^*P* < 0.0005, ^∗∗∗∗^*P* < 0.0001.

FIGURE S2 The effects of different concentrations of IFNα-2b on HBV replication and transcription in HepG2.2.15 cell line. HBV replication intermediates were extracted and detected by Southern blot hybridization **(A)** and quantified using fluorescence quantitative PCR **(B)**. Viral RNAs were examined by Northern blot **(C)** and quantified using fluorescence quantitative PCR **(D)**. All values are expressed as percentages relative to the untreated control. RC, relaxed circular DNA; SS, single-stranded DNA. β-actin served as the DNA loading control and 18S/28S RNAs served as the RNA loading control. Statistical significance was calculated using student’s *t*-test. Ns, not significant, ^∗^*P* < 0.05, ^∗∗^*P* < 0.005, ^∗∗∗^*P* < 0.0005, ^∗∗∗∗^*P* < 0.0001.

FIGURE S3 Lettuce extracts S15 suppresses HBV viral antigen **(A,B)**, replication **(C,D)** and transcription **(E,F)** in the absence or presence 3TC and IFNa-2b in HepAD38 cells. HBsAg and HBeAg levels in culture supernatants were measured using an electrochemical illuminescent immunoassay **(A,B)**. HBV replication intermediates were extracted and detected by Southern blot hybridization **(C)** and quantified using fluorescence quantitative PCR **(D)**. Viral RNAs were examined by Northern blot **(E)** and quantified using fluorescence quantitative PCR **(F)**. All values are expressed as percentages relative to untreated control. RC, relaxed circular DNA; SS, single-stranded DNA. β-actin served as the DNA loading control and 18S/28S RNAs served as the RNA loading control. Statistical significance was calculated using student’s *t*-test. Ns, not significant, ^∗^*P* < 0.05, ^∗∗^*P* < 0.005, ^∗∗∗^*P* < 0.0005, ^∗∗∗∗^*P* < 0.0001.

## Abbreviations

3TC: lamivudine
CHB: chronic hepatitis B
CsA: cyclosporine A
DAPI: 4′,6-diamidino-2-phenylindole
DCFH-DA: 2′, 7′-dichlorofluorescin diacetate
DMEM: Dulbecco’s modified Eagle’s medium
ER: endoplasmic reticulum
FBS: fetal bovine serum
HBeAg: hepatitis B e antigen
HBsAg: hepatitis B surface antigen
HBV: hepatitis B virus
IFNα-2b: interferon-alpha 2b
JC-1: 5,5′,6,6′-tetrachloro-1,1′,3,3′-tetraethylbenzimidazolylcarbocyanine iodide
LdT: telbivudine
MitoRed: 9-[2-(4′-Methylcoumarin-7′-oxycarbonyl) phenyl]-3,6-bis(diethylamino) xanthylium chloride
NA: nucleoside analog
NAC: *N*-acetyl-L-cysteine
NIM811: *N*-Methyl-4-isoleucine cyclosporine
pgRNA: pregenomic RNA
Q-PCR: real-time fluorescence quantitative PCR
RI: replicative intermediates
ROS: reactive oxygen species
UPLC/IMS/QTOF-MS: ultra-performance liquid chromatography ion mobility spectrometry quadrupole time of flight mass spectrometer
