# Supplementary figures and images for: Luteolin-7-O-Glucoside Present in Lettuce Extracts Inhibits Hepatitis B Surface Antigen Production and Viral Replication by Human Hepatoma Cells in Vitro
Source: Front Microbiol. 2017 Dec 6;8:2425. doi: 10.3389/fmicb.2017.02425 (PMC5723679; doi:10.3389/fmicb.2017.02425)

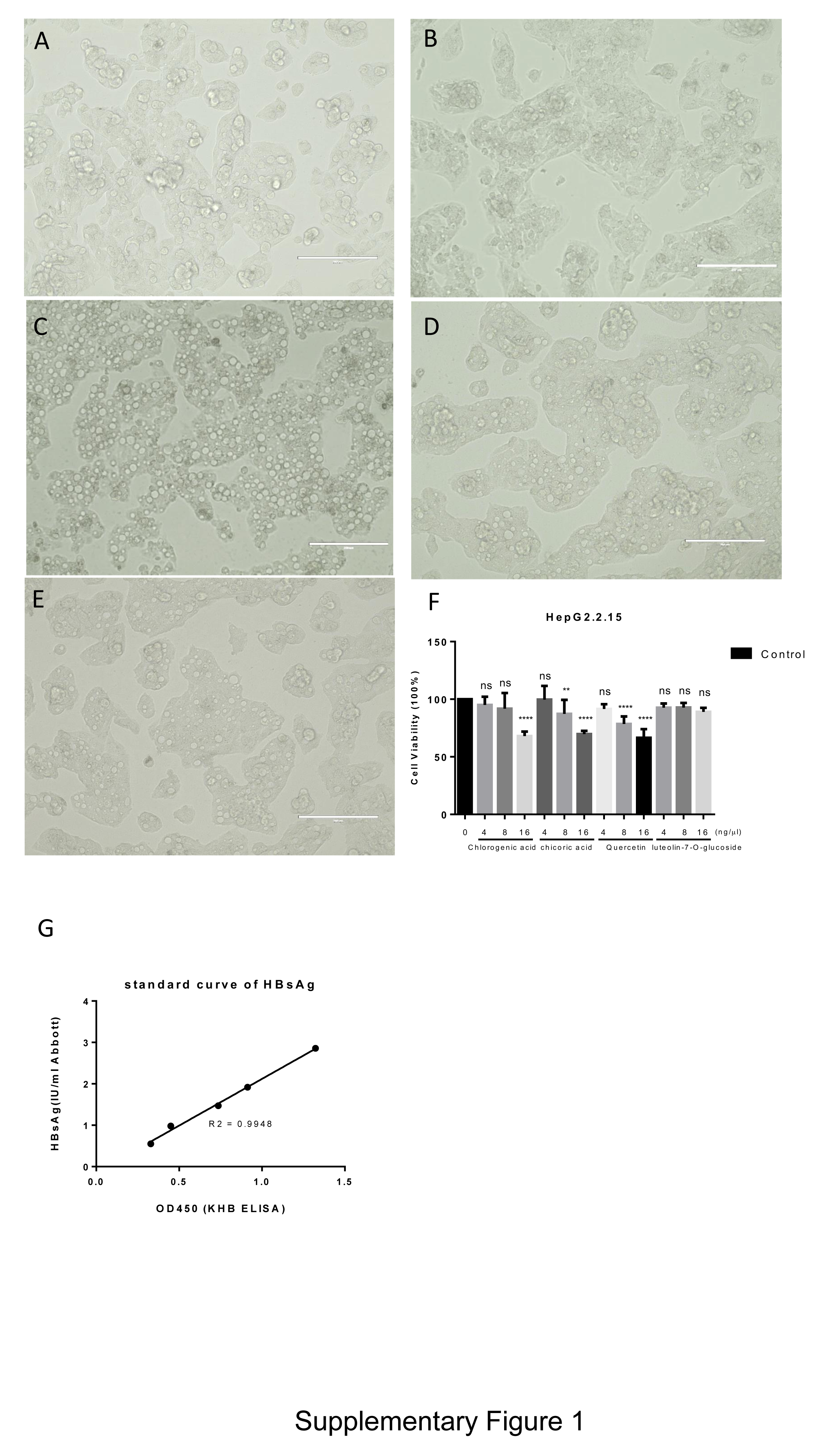

Supplement: FIGURE S1 — Cytotoxicity of lettuce extracts and differential metabolites in HepG2.2.15 cells. (A) Represented the morphology of normal cells and (B) represented the morphology of the cells that treated with luteolin-7-O-glucoside. A large number of vacuoles were observed in cells treated with quercetin-3-(6″-malonylglucoside) (C), chicoric acid (D) and chlorogenic acid (E) at the concentrations. The CCK8 assay provided consistent results as the morphological analysis (F). Semi-quantitative analysis of HBsAg and validation of the HBsAg ELISA standard curve using a commercial quantitative assays (G). Statistical significance was calculated using student’s t-test. Ns, not significant, ∗P < 0.05, ∗∗P < 0.005, ∗∗∗P < 0.0005, ∗∗∗∗P < 0.0001. [file Image_1.TIF]

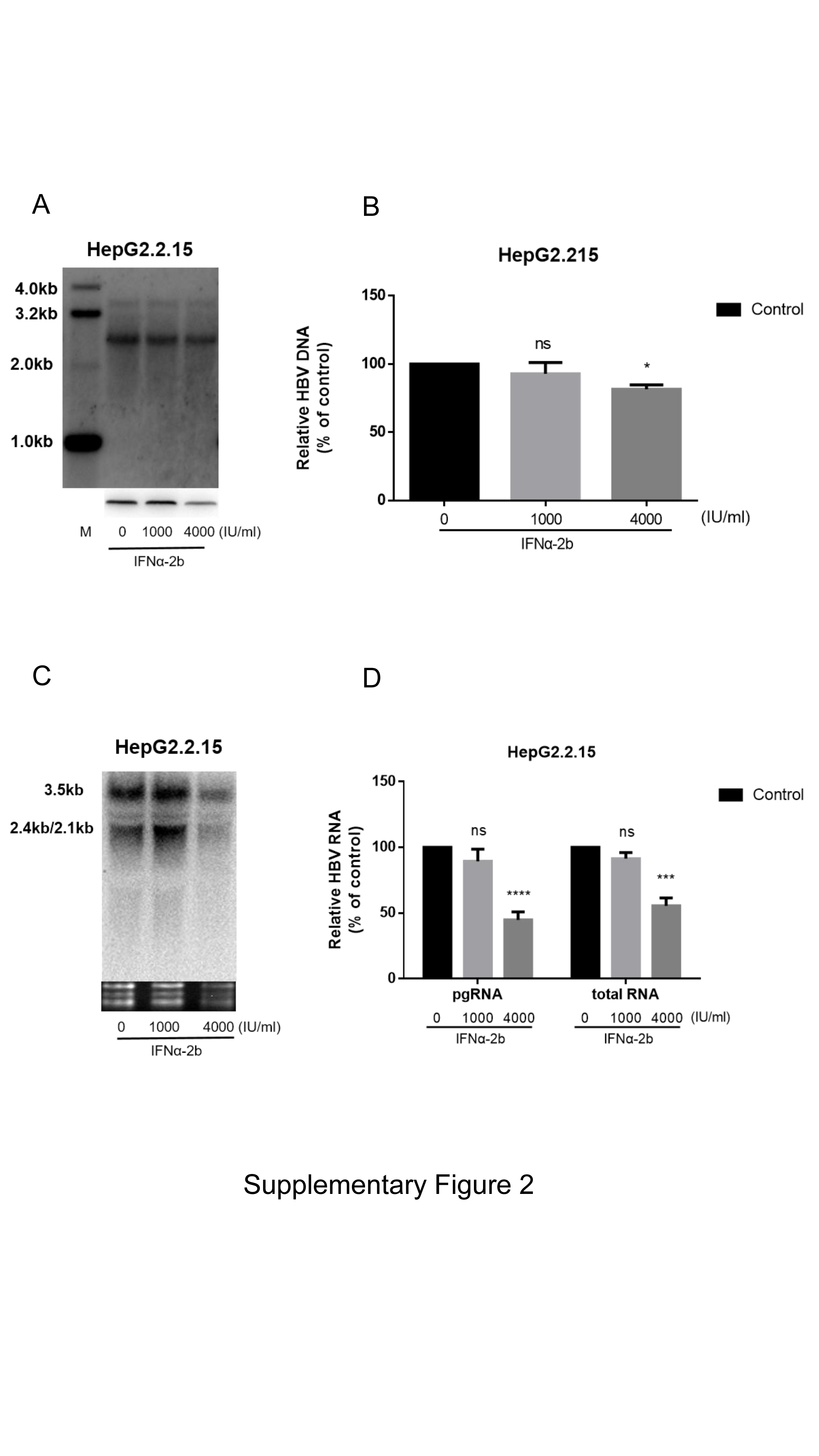

Supplement: FIGURE S2 — The effects of different concentrations of IFNα-2b on HBV replication and transcription in HepG2.2.15 cell line. HBV replication intermediates were extracted and detected by Southern blot hybridization (A) and quantified using fluorescence quantitative PCR (B). Viral RNAs were examined by Northern blot (C) and quantified using fluorescence quantitative PCR (D). All values are expressed as percentages relative to the untreated control. RC, relaxed circular DNA; SS, single-stranded DNA. β-actin served as the DNA loading control and 18S/28S RNAs served as the RNA loading control. Statistical significance was calculated using student’s t-test. Ns, not significant, ∗P < 0.05, ∗∗P < 0.005, ∗∗∗P < 0.0005, ∗∗∗∗P < 0.0001. [file Image_2.TIF]

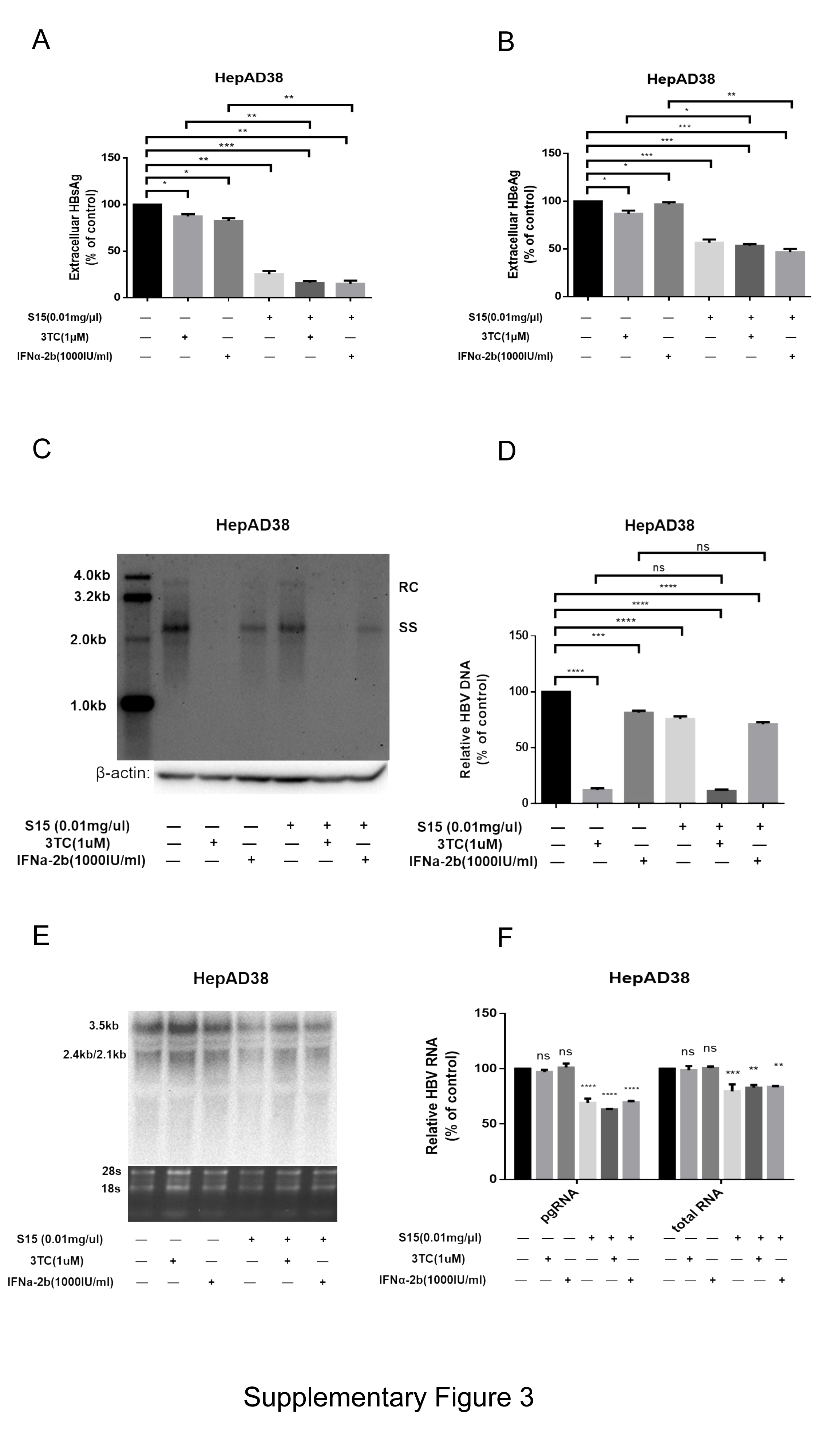

Supplement: FIGURE S3 — Lettuce extracts S15 suppresses HBV viral antigen (A,B), replication (C,D) and transcription (E,F) in the absence or presence 3TC and IFNa-2b in HepAD38 cells. HBsAg and HBeAg levels in culture supernatants were measured using an electrochemical illuminescent immunoassay (A,B). HBV replication intermediates were extracted and detected by Southern blot hybridization (C) and quantified using fluorescence quantitative PCR (D). Viral RNAs were examined by Northern blot (E) and quantified using fluorescence quantitative PCR (F). All values are expressed as percentages relative to untreated control. RC, relaxed circular DNA; SS, single-stranded DNA. β-actin served as the DNA loading control and 18S/28S RNAs served as the RNA loading control. Statistical significance was calculated using student’s t-test. Ns, not significant, ∗P < 0.05, ∗∗P < 0.005, ∗∗∗P < 0.0005, ∗∗∗∗P < 0.0001. [file Image_3.TIF]
